# Lighting the Mouth

**Published:** 1896-06

**Authors:** William H. Rollins

**Affiliations:** Boston, Mass.


### LIGHTING THE MOUTH.

BY WILLIAM H. ROLLINS, BOSTON, MASS.

THE arc light is the best artificial light for the mouth, and even in good daylight it improves the seeing in filling difficult cavities. The cut, which is taken from a photograph, shows a good way to arrange this light, as is proved by my having used this apparatus without change for three years.

